# Hepatic Stellate Cells and Hepatocytes as Liver Antigen-Presenting Cells during *B. abortus* Infection

**DOI:** 10.3390/pathogens9070527

**Published:** 2020-06-30

**Authors:** Paula Constanza Arriola Benitez, Ayelén Ivana Pesce Viglietti, María Mercedes Elizalde, Guillermo Hernán Giambartolomei, Jorge Fabián Quarleri, María Victoria Delpino

**Affiliations:** 1 Instituto de Inmunología, Genética y Metabolismo (INIGEM), Universidad de Buenos Aires, CONICET, Buenos Aires 1120, Argentina; constanza-arriola@hotmail.com (P.C.A.B.); ayelenpv@gmail.com (A.I.P.V.); ggiambart@ffyb.uba.ar (G.H.G.); 2 Instituto de Investigaciones Biomédicas en Retrovirus y Sida (INBIRS), Universidad de Buenos Aires, CONICET, Buenos Aires 1121, Argentina; mecheeli@hotmail.com

**Keywords:** *Brucella*, HSC, MHC, IL-10

## Abstract

In Brucellosis, the role of hepatic stellate cells (HSCs) in the induction of liver fibrosis has been elucidated recently. Here, we study how the infection modulates the antigen-presenting capacity of LX-2 cells. *Brucella abortus* infection induces the upregulation of class II transactivator protein (CIITA) with concomitant MHC-I and -II expression in LX-2 cells in a manner that is independent from the expression of the type 4 secretion system (T4SS). In concordance, *B. abortus* infection increases the phagocytic ability of LX-2 cells and induces MHC-II-restricted antigen processing and presentation. In view of the ability of *B. abortus*-infected LX-2 cells to produce monocyte-attracting factors, we tested the capacity of culture supernatants from *B. abortus*-infected monocytes on MHC-I and –II expression in LX-2 cells. Culture supernatants from *B. abortus*-infected monocytes do not induce MHC-I and -II expression. However, these supernatants inhibit MHC-II expression induced by IFN-γ in an IL-10 dependent mechanism. Since hepatocytes constitute the most abundant epithelial cell in the liver, experiments were conducted to determine the contribution of these cells in antigen presentation in the context of *B. abortus* infection. Our results indicated that *B. abortus*-infected hepatocytes have an increased MHC-I expression, but MHC-II levels remain at basal levels. Overall, *B. abortus* infection induces MHC-I and -II expression in LX-2 cells, increasing the antigen presentation. Nevertheless, this response could be modulated by resident or infiltrating monocytes/macrophages.

## 1. Introduction

*Brucella* spp. are Gram-negative intracellular bacteria that infect domestic and natural animals and produce an incapacitating chronic disease when transmitted to humans. In many countries, brucellosis remains endemic. The most frequent clinical characteristics are hepatomegaly, splenomegaly and peripheral lymphadenopathy, revealing the preference of *Brucella* for the reticuloendothelial system [1,2].

As a frequent niche of infections, the liver provides a tolerogenic environment. Such immunotolerant capacity is based on the presence of a resident immune cell repertoire in constant stimulation and the hepatic blood source that spread a unique growth factor and cytokine milieu [3].

However, the immune system of the liver is capable of inducing a prompt-response to tumor cells and pathogenic microorganisms [4]. Thus, the majority of the microorganisms that arrive in the liver are eradicated. Nonetheless, even though these several mechanisms can remove infectious agents, *Brucella* spp. can escape the immune response and persist in the liver. Accordingly, in humans infected with *Brucella*, the liver is frequently implicated, with a frequency between 5% to 52% or more [5]. Liver biopsies from *Brucella abortus*-infected patients revealed the presence of granulomas with single of multiple localizations in portal and parenchymal tissue, inflammatory infiltrations, and parenchymal necrosis [6,7].

Among the non-parenchymal cells, hepatic stellate cells (HSCs) are placed among hepatocyte and small blood vessels. They are characterized by their contents of intracellular lipid droplets and protuberances that spread nearby the blood vessels. During liver injury, HSCs are activated and realize collagen with the development of scar tissue, producing chronic fibrosis or cirrhosis [8]. Furthermore, they also have a role in liver fibrosis to heal restore inflammatory injury.

During *B. abortus* infection, the protagonism of HSCs during the generation of fibrosis has recently been revealed [9]. Besides their function during liver damage through the production of fibrosis, HSCs cans also participate as local antigen-presenting cells (APCs). HSCs express MHC class I and II molecules, as well as co-stimulatory molecules such as CD40 and CD80 [10]. Accordingly, HSCs can interact with CD4^+^ T cells to induce effector responses [11]. In addition, HSCs direct naïve CD4^+^ T-cell activation to T_reg_ differentiation in the presence of Dendritic cells (DC) [12]. Thus, the main role of HSCs is the ability to induce a tolerogenic liver milieu that can favor the chronicity of *B. abortus* infection.

Nucleated cells express MHC class I molecules, but MHC class II molecule expression is restricted for cell types such as dendritic cells, macrophages and B lymphocytes. MHC class II expression is regulated in part by the class II transactivator protein (CIITA) at the transcription level. The α- and β-chains of newly synthesized class II molecules are associated with the invariant change (Ii), giving rise to immature MHC-II. These molecules reach the cell surface, then recycle to the endosomal/lysosomal compartment, named MIIC. In this compartment, cathepsin S is one of the proteases responsible in Ii processing to Class II-associated invariant chain peptide (CLIP) in human antigen-presenting cells. Ii removal is an important step for the adequate export of the peptide-loaded class II molecule to the cell surface. Activation of HSCs by several agonists such as bacterial lipopolysaccharide (LPS) and IFN-γ drive the increase of MHC class II expression and co-stimulatory molecules [11]. Immune responses to liver pathogens need to consider the possibility that unconventional Antigen presenting cells (APC) play an important function, and may account for the miscarriage of effective immunity. Thus, the aim of this study is to characterize the induction of surface MHC-I and -II expression during *B. abortus* infection.

## 2. Results

### 2.1. B. abortus Infection Induces MHC-I and -II Expression in LX-2 Cells via a T4SS-Independent Mechanism

**Figure 1:**
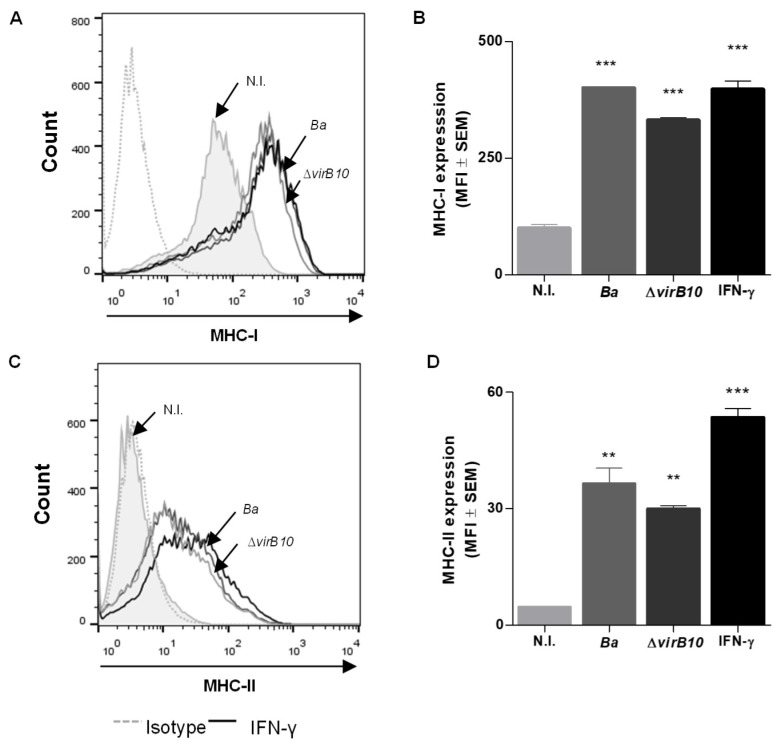
*Brucella abortus* infection induces MHC-I and -II expression in LX-2 cells. LX-2 cells were infected with *B. abortus* or *B. abortus virB10* mutant (Δ*virB10*) at a multiplicity of infection (MOI) of 1000 for 2 h, washed, and incubated for 72 h in complete media with antibiotics. IFN-γ (5oo U/mL) was used as a positive control. Non-infected cells (N.I.). MHC-I (**A**,**B**) and MHC-II (**C**,**D**) expression was assessed by flow cytometry. The histograms indicate the results of one representative of five independent experiments (**A**,**C**). The bars indicate the arithmetic means of five experiments, and the error bars indicate the standard errors of the means. MFI, mean fluorescence intensity (**B**,**D**). Non-specific binding was determined using a control isotype (Isotype). **, *p* < 0.01; ***, *p* < 0.001 versus non-infected cells (N.I.).

In this section, the capacity of *B. abortus* to induce the expression of MHC-I and -II molecules on LX-2 cells is determined. Cells were infected with *B. abortus* for 2 h, washed to eliminate the bacteria, and the infection was continued for additional 72 h. Our results indicate that *B. abortus* infection stimulated MHC-I and -II expression in LX-2 cells, yielding a level comparable to that of IFN-γ-stimulated cells used as a positive control (Figure 1).

The type 4 secretion system (T4SS) encoded by the *vir*B operon participates in the establishment of the intracellular replication niche of *Brucella* in different cell types [13], as well as contributing to the induction of a fibrotic phenotype in HSCs during *B. abortus* infection. We decided to test whether MHC-I and -II expression in infected LX-2 cells depends on a functional T4SS. No significant difference in MHC-I and -II expression was found between LX-2 cells infected with the wild-type strain and those infected with *vir*B10 isogenic mutants, indicating that the T4SS is not implicated in the modulation of MHC-I and -II expression (Figure 1). These results indicate that *B. abortus* infection induces MHC-I and -II upregulation in a T4SS-independent mechanism.

### 2.2. B. abortus Infection Induce CIITA and Cathepsin S Transcription in LX-2 cells

**Figure 2:**
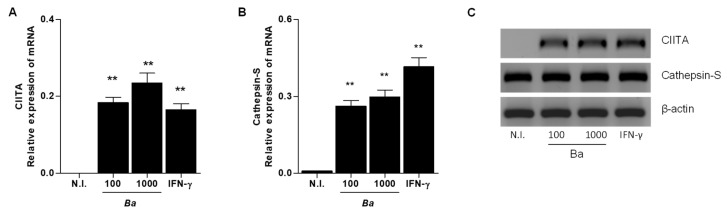
*B. abortus* infection induces Class II Major Histocompatibility Complex Transactivator (CIITA) and cathepsin-S expression in LX-2 cells. LX-2 cells were infected with *B. abortus* (*Ba*) at (MOIs) of 100 and 1000. At 72 h, post-infection levels of CIITA (**A**) and cathepsin-S (**B**) were determined by RT-qPCR. Agarose gel of PCR products from endpoint PCR products (**C**). Data are given as the means ± SD from three individual experiments. **, *p* < 0.01; ***, *p* < 0.001 versus non-infected cells (N.I.).

At transcription level, CIITA plays a key role in the MHC class II expression in professional antigen-presenting cells (APCs). Thus, experiments were conducted to evaluate whether increasing MHC-II expression correlated with increased transcription of CIITA. *B. abortus* infection induced up-regulation of CIITA mRNA after 72 h post-infection. IFN-γ was used as positive control (Figure 2A). The maturational processing of the MHC-II required the cleavage of li. The main protease involved in this process is cathepsin S [14]. Then, experiments were conducted to determine whether *B. abortus* infection was able to induce cathepsin S upregulation. *B. abortus* up-regulated cathepsin S mRNA at 72 h post-infection. IFN-γ was used as a positive control (Figure 2B). CIITA and cathepsin S primer specificity were determined by endpoint PCR (Figure 2C). These results indicate that *B. abortus* infection induces CIITA and cathepsin S transcription accordingly with the increase of MHC-II.

### 2.3. B. abortus Infection Does not Induce the Expression of Costimulatory Molecules CD80, CD86 and CD40

For T cells, activation the recognition of antigen/MHC complex by the T cell receptor (TCR) must be complemented by a second signal that is provided by costimulatory molecules. Experiments were conducted to determine whether *B. abortus* infection could induce CD80, CD86 and CD40 expression on LX-2 cells. *B. abortus* was unable to induce costimulatory molecule expression measured at 72 h post-infection by using specific antibodies (not shown). These results indicated that even though *B. abortus* was able to induce MHC upregulation, the costimulatory molecules remained at basal levels.

### 2.4. B. abortus Increase the Phagocytic Capability of LX-2 Cells

**Figure 3:**
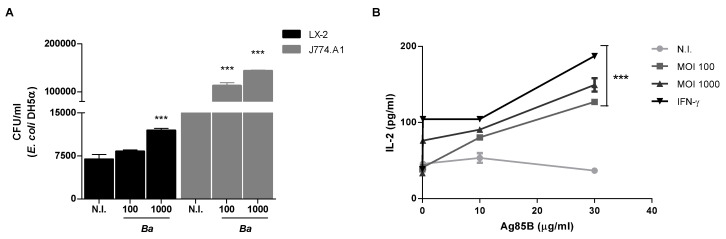
*B. abortus* increased the phagocytic capability and induced MHC-II-restricted processing and presentation in LX-2 cells. LX-2 cells were infected with *B. abortus* (*Ba*) at MOIs of 100 and 1000. After 72 h post-infection, *Escherichia coli* was added to the culture. Phagocytized *E. coli* were evaluated by intracellular colony-forming unit (CFU) counting (**A**); or after 72 post-infection, cells were pulsed with Ag85B for 6 h, followed by incubation with DB1 cells for 24 h. Supernatants were harvested and the amount of IL-2 was determined by ELISA (**B**). Data are given as the means ± SD from five individual experiments. ***, *p* < 0.001 versus non-infected cells (N.I.).

To determine whether *B. abortus* infection increase the phagocytic ability of LX-2 cells, cells were infected with *B. abortus* for 24 h then cultured with *Escherichia coli* for 30 min. Antibiotics were added to kill non-phagocyted *E. coli*. Counting of colony-forming unit (CFU) was performed to determine the phagocyted bacteria. *B. abortus*-infected LX-2 cells at an MOI of 1000 significantly increased phagocytosed *E. coli* in relation to uninfected cells (Figure 3A). We compared the phagocytic capacity of LX-2 cells with respect to the macrophage cell line J774.A1. Uninfected J774.A1 cells had an increased phagocytic capacity with respect to uninfected LX-2 cells. In addition, phagocytosed *E. coli* was significantly increased when J774.A1 cells were infected with *B. abortus* in an MOI-dependent fashion (Figure 3A). These results indicate that *B. abortus* infection increases the phagocytic capacity of LX-2 cells. However, its phagocytic capacity was lower than that observed in macrophages.

### 2.5. B. abortus Induces MHC-II-Restricted Antigen Processing and Presentation by LX-2 Cells

To determine if the MHC-II upregulation promoted by *B. abortus* infection was related to changes in antigen processing and the presentation of soluble antigens for MHC-II-restricted T cells, LX-2 cells were infected for 72 h then incubated with Ag85B from *Mycobacterium tuberculosis* and DB1 T-cell hybridoma, which identify soluble Ag85B processed and presented by LX-2 cells (HLA-DR1). The infection with *B. abortus* significantly increased antigen processing and presentation at multiple Ag85B concentrations, as was revealed by the increased amount of IL-2 produced by T-cell hybridoma (Figure 3B). Thus, *B. abortus* infection induces processing and presentation of soluble antigens by LX-2 cells.

### 2.6. Culture Supernatants from B. abortus Infected THP-I Cells Do not Induce MHC-I and MHC-II Expression by LX2 Cells

**Figure 4:**
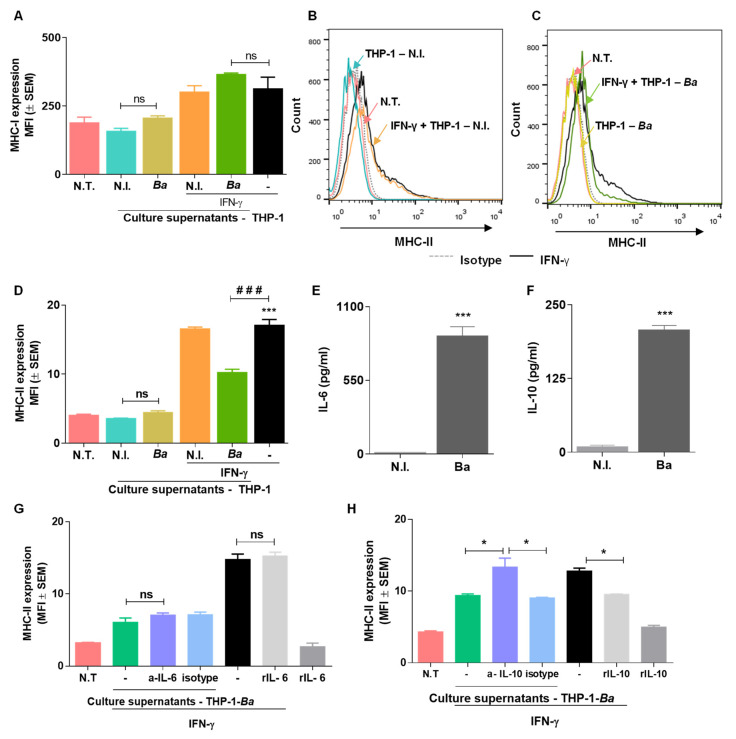
Modulation of MHC-I and -II expression in LX-2 cells by culture supernatants from *B. abortus*-infected monocytes. LX-2 cells were stimulated with culture supernatants from THP-1 cells infected at an MOI of 100 in the presence or not of IFN-γ (500 U/mL) or culture supernatants from uninfected THP-1 cells as control at a 1/2 dilution. After 72 h post-stimulation, MHC-I (**A**), MHC-II (**B**–**D**), expression was assessed by flow cytometry. IL-6 and IL-10 were determined by ELISA in culture supernatants from *B. abortus*-infected THP-1 cells at a MOI of 100 (**E**,**F**). MHC-II expression in LX-2 treated with culture supernatants from infected THP-1 cells plus IFN-γ was assayed in the presence of a neutralizing antibody anti-IL-6 (20 µg/mL), anti-IL-10 (20 ug/mL), or their isotype-matched control, with 10 ng/mL of recombinant human IL-6 (rIL-6) or 10 ng/mL of recombinant human IL-10 (rIL-10) alone or plus IFN-γ used as a control (**G**,**H**). The histograms indicate the results of one representative of five independent experiments (**A**–**D**,**G**,**H**). The bars indicate the arithmetic means of five experiments, and the error bars indicate the standard errors of the means. MFI, mean fluorescence intensity (**A**,**D**). Non-specific binding was determined using a control isotype (Isotype). *, *p* < 0.1; **, *p* < 0.01; ***, *p* < 0.001 versus non-infected cells (N.I.) and cells stimulated with culture supernatants from uninfected THP-1 cells.

In view of the capacity of *B. abortus*-infected LX-2 cells to produce chemoattractant factors of a monocyte [9] that could attract monocytes to the site of infection, we evaluated whether supernatants from *B. abortus*-infected THP-1 cells were able to modulate MHC-I and -II in LX-2 cells. To this end, LX-2 cells were treated with a 1/2 dilution of supernatants from *B. abortus*-infected and uninfected monocytes over 72 h. IFN-γ was used as a positive control. Supernatants from *B. abortus*-infected monocytes did not alter the MHC-I and -II expression levels in LX-2 cells (Figure 4A–D).

### 2.7. Culture Supernatants from B. abortus Infected THP-I Monocytes Inhibit MHC-II Expression Induced by IFN-γ in an IL-10 Dependent Mechanism. 

After *B. abortus* infection of macrophages, their IFN-γ-induced expression of MHC-I and -II molecules is inhibited [15,16]. Here, we have demonstrated that the *B. abortus* infection of macrophages also hits the IFN-γ-induced MHC-II but not the MHC-I expression in HSC cells (Figure 4A–D). IL-6 and IL-10 were found to be involved in the inhibition of MHC-II in different cell types, including during *B. abortus* infection [15,17], and THP-1 cells were found to secrete IL-6 and IL-10 in response to *B. abortus* infection (Figure 4E,F). Moreover, when the IL-6 and IL-10 involvement was assessed using the neutralization of specific antibodies, IL-10 but not IL-6 participated in MHC-II downregulation in LX-2 cells. In addition, IL-10 present in culture supernatants from THP-1 cells was involved in the inhibition of MHC-II expression induced by IFN-γ, since a neutralizing antibody (anti IL-10) was able to reverse the inhibitory effect (Figure 4H). In contrast, recombinant IL-6 did not inhibit MHC-II expression induced by IFN-γ in LX-2 cells (Figure 4G).

### 2.8. B. abortus Infection Induces MHC-I Expression in HepG2 Cells but Does not Alter MHC-II Levels

**Figure 5:**
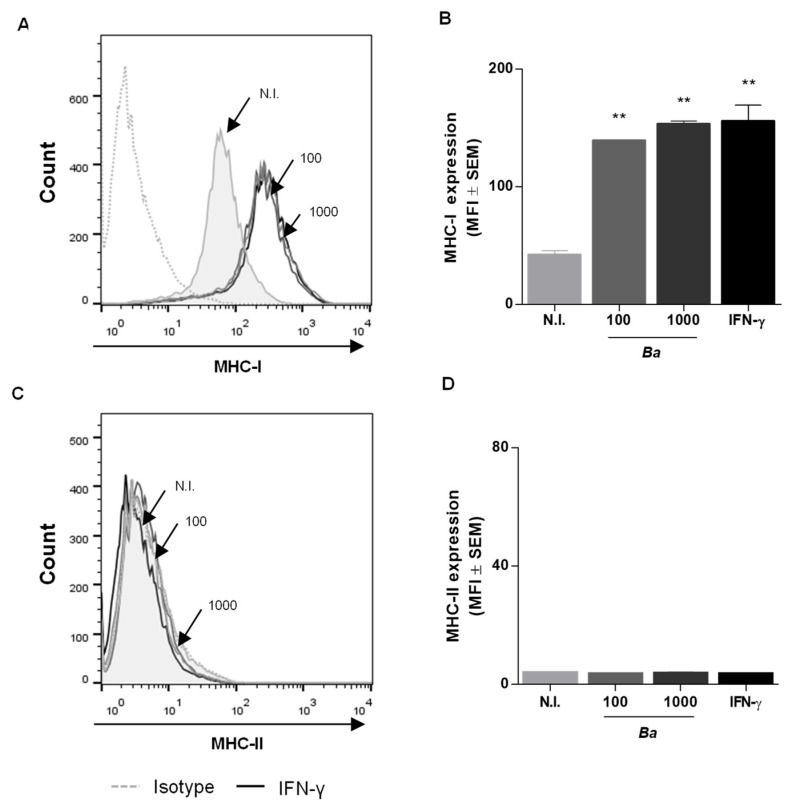
*B. abortus* infection induces MHC-I expression in HepG2 cells but does not alter MHC-II levels. HepG2 cells were infected with *B. abortus* at multiplicity of infections (MOIs) of 100 and 1000 for 2 h, washed, and incubated for 72 h in complete media with antibiotics. IFN-γ (500 U/mL) was used as a positive control. MHC-I (**A**,**B**) and MHC-II (**C**,**D**) expression were assessed by flow cytometry. The histograms indicate the results of one representative of five independent experiments (**A**,**C**). The bars indicate the arithmetic means of five experiments, and the error bars indicate the standard errors of the means. MFI, mean fluorescence intensity (**B**,**D**). Non-specific binding was determined using a control isotype (Isotype). **, *p* < 0.01 versus non-infected cells (N.I.).

Previously we have demonstrated that *B. abortus* infects and replicates in HepG2 hepatocytes [18]. These cells represent around 60% of liver mass, and do not express MHC-II molecules under physiological conditions. However, under inflammatory conditions, hepatocytes can express MHC-II molecules and also activate T cells [19]. Experiments were conducted to determine if *B. abortus* infection is capable of modulating MHC-II expression in HepG2 hepatocytes. To this end, HepG2 cells were infected with *B. abortus* and at 72 h post-infection, and the expression of MHC-I and -II molecules were measured. *B. abortus* infection was able to differentially induce MHC-I but not MHC-II expression (Figure 5). In addition, IFN-γ was unable to induce MHC-II expression in HepG2 cells (Figure 5B).

## 3. Discussion

Most of the microorganisms that arrive in the liver are eliminated due to the balance between tolerance and inflammation of the hepatic microenvironment [3,20,21]. *Brucella* has a panoply of defensive strategies to evade immune response, including intracellular lifestyle and the prevention of the development of an appropriate adaptive immune response [22,23]. Thus, *Brucella* escapes from the immune response and persists in the liver, as demonstrated by the high frequency of liver pathology in human disease [5]. HSCs depict a pivotal function for wound healing of the liver [24]. Notwithstanding, the antigen-presenting capacity of HSCs has been previously reported in studies that revealed the expression of basal levels of costimulatory molecules and increases in MHC-II expression in response to IFN-γ [11,25,26]. In this study, we demonstrated the ability of *B. abortus* infection of HSCs (LX-2 cells) to upregulate MHC-I and -II expression, while the expression of the costimulatory molecules (CD80, CD86 and CD40) remained at basal levels.

The antigen presentation process involves recognition, uptake and processing by antigen-presenting cells. Previously, it has been described that the uptake of antigens by HSC is less effective than other professional APCs [27]. However, it is known that mature dendritic cells have a poor endocytic capacity, but effectively present antigens to T cells [28]. Nevertheless, *B. abortus* infection increased the efficiency of antigen uptake significantly via HSCs. Moreover, when these HSC-infected cells were cocultured with T cells, a higher level of IL-2 secretion was measured, thus inferring an increased antigen processing and further MHC-II-restricted T cell presentation after *B. abortus* infection. These results opposed other studies that have indicated that HSCs not only do not induce an effective T-cell response, but also induce the apoptosis of T cells through B7-H1 and B7-H4 signaling [26,29,30]. Such a discrepancy could be attributed to the fact that these studies eliminated the uptake, processing and presentation of antigens, since the T cell responses were performed by peptide pulsed-HSCs.

In B cells, thymus epithelial cells, and myeloid dendritic cells, CIITA is the master regulator of major histocompatibility complex (MHC) gene expression, which is constitutively expressed. However, in HSCs (among several cell types), the transcription of CIITA requires IFN-γ among others factors for both MHC-II expression [31,32] and the modulation of the transcription of MHC-I genes [33,34,35]. Accordingly, when HSCs are infected by *B. abortus*, the expression of MHC-I and -II are upregulated.

Antigen processing and presentation require several lysosomal proteases, including cathepsin B, L, D, and S, which are involved in the maturation of MHC-II through the processing of Ii and the cleavage of antigen peptides that will be presented [36,37,38,39]. However, the most effective proteases involved in the last step of the Ii cleavage process are cathepsin S and L. Depending on the cell type, cathepsin L and S are involved in peptide degradation [39,40]. Recently, it has been shown that cathepsin S is expressed in HSCs, which can be induced by proinflammatory cytokines such as IFN-γ. This suggests a contribution to Ii processing. In contrast, cathepsin L expression has not been significantly increased at the transcription level upon stimulation with IFN-γ [41], indicating that cathepsin S has a central role in antigen presentation in HSCs. In accordance with the increase in MHC-II expression, antigen processing, and presentation in MHC-II restricted T cells, *B. abortus* infection has also been able to induce cathepsin S mRNA transcription in HSCs.

The T4SS encoded by *virB* genes has been involved in the ability of *Brucella* to begin its intracellular replication niche [22]. In HSCs, we have previously reported that the T4SS is required to induce inflammasome activation and a fibrotic phenotype during *B. abortus* infection [42]. This system has been found to participate in the stimulation of inflammatory response during *B. abortus* infection both in vivo and in vitro [43]. However, our experiments using an isogenic a *B. abortus vir*B10 polar mutant indicated that the T4SS was not involved in the induction of MHC-I and -II expression stimulated by *B. abortus* infection in HSCs.

The virulence of *B. abortus* relies on the ability of this bacteria to interact with macrophages as a central event for launching chronic *Brucella* infections [44,45]. In previous studies we have demonstrated that HSCs secrete MCP-1 in response to *B. abortus* infections [9], indicating that monocytes/macrophages could be attracted to the site of infection and, in conjunction with the resident macrophages, could modulate HSC responses. However, our results indicate that supernatants from *B. abortus*-infected macrophages were unable to induce MHC-I and -II expression.

*B. abortus* infection has been shown to potently activate a proinflammatory response that triggers the differentiation of T-cell responses to T-helper 1 (Th1) [46] with the simultaneous production of IFN-γ [47]. This cytokine enhances not only microbicide activities of macrophages, but also antigen-presenting functions in cells [48]. However, *B. abortus* infection can stimulate not only inflammatory but also immunomodulatory mediators such as IL-10 and IL-6 through monocytes [49,50]. These cytokines have been reported as responsible for inhibiting IFN-γ-induced MHC-II expression in immune cells [51,52]. Our experiments demonstrate that during the *B. abortus* infection of HSCs, IL-10 but not IL-6 present in supernatants from *B. abortus*-infected monocytes was implicated, at least in part, in the inhibition of IFN-γ-induced MHC-II expression.

*B. abortus* infection can infect and replicate in hepatocytes, inducing an inflammatory response [18]. Here, we demonstrate that in the setting of *B. abortus* infection, the MHC-I but not the MHC-II expression was induced in hepatocytes, thus enabling the hepatocytes to be susceptible to CD8+ cytotoxic T cell action.

In conclusion, the *B. abortus* infection of hepatic stellate cells and hepatocytes is able to regulate differentially the MHC expression, thus stimulating the T-cell specific-immune response at the liver. However, due to a cellular interplay, such responses may also be modified by resident or infiltrating *B. abortus*-infected monocytes/macrophages. Such bacterial skills exerted on hepatic cells may promote the evasion of immune surveillance, thus favoring its chronicity in the liver.

## 4. Materials and Methods

### 4.1. Bacterial Culture

*Brucella abortus* S2308 or the isogenic *B. abortus vir*B10 polar mutant (kindly provided by Diego Comerci, UNSAM University, Argentina) were cultivated in 10 ml of tryptic soy broth (Merck, Buenos Aires, Argentina) for 18 h with constant agitation at 37 °C. Bacteria were harvested and the inoculum were prepared as described previously [53]. All experiments with live *Brucella* were carried out in biosafety level 3 facilities located at the Instituto de Investigaciones Biomédicas en Retrovirus y SIDA (INBIRS).

### 4.2. Cell Culture

The spontaneously immortalized human hepatic stellate cell line (LX-2) was kindly provided by Dr. Scott L. Friedman (Mount Sinai School of Medicine, New York, NY, USA). LX-2 cells were maintained in Dulbecco’s Modified Eagle Medium (DMEM, Life Technologies, Grand Island, NY, USA) and supplemented with 5% fetal bovine serum (FBS; Life Technologies), L-glutamine (2 mM), sodium pyruvate (1 mM), 100 U/mL penicillin, and 100 µg/mL streptomycin. The human hepatoma cell line HepG2, the murine J774.A1 cell line, and the human monocytic cell line THP-1 were obtained from the ATCC (Manassas, VA, USA) and were cultured as previously described [18]. Monocyte differentiation from THP-1 cells was achieved through cultivation in the presence of 0.05 mmol/L 1, 25-dihydroxyvitamin D3 (Calbiochem-Nova Biochem International, La Jolla, CA, USA) for 72 h. DB1 T hybridoma cells (Ag85B specific) was kindly provided by W. H. Boom (Case Western Reserve University, Cleveland, OH, USA) and was maintained in DMEM supplemented as indicated above. All cultures were grown at 37 °C and 5% CO_2_.

### 4.3. Cellular Infection

LX-2 cells were dispensed in 24-well plates and infected with *B. abortus* S2308 or *B. abortus virB10* polar mutant at a multiplicity of infection (MOI) of 100 or 1000. HepG2 cells were infected with *B. abortus* S2308 at an MOI of 100 or 1000, and THP-1 cells at an MOI of 100. After the bacterial suspension was dispensed, the plates were centrifuged for 10 min at 2000 rpm, then incubated for 2 h at 37 °C under a 5% CO_2_ atmosphere. To remove extracellular bacteria, Cells were extensively washed with DMEM then incubated in medium supplemented with 100 µg/mL gentamicin and 50 µg/mL streptomycin to kill extracellular bacteria. LX-2 cells were harvested at 72 h to determine major histocompatibility complex class I (MHC-I), MHC-II, CD40, CD80, and CD86 surface expression and CIITA and cathepsin-S gene expression. Supernatants from THP-1 cells were harvested 24 h after infection to be used as conditioned medium.

### 4.4. Flow Cytometry

Infected LX-2 cells, cells treated with culture supernatants at a 1/2 dilution from THP-1 cells, or recombinant human IFN-γ-treated-LX-2 cells (500 U/mL; Endogen) were washed and incubated with fluorescein isothiocyanate-labeled (FITC) anti-human HLA-DR monoclonal antibody (MAb) (clone L243; BD Bioscience, San Diego, CA, USA), FITC-labeled anti-human HLA-ABC (clone G46-2.6; BD Bioscience), phycoerythrin (PE)-labeled anti-human CD40 (clone 5C3; BD Bioscience), PE-labeled anti-human CD86 (clone 2331(FUN-1); BD Bioscience) FITC-labeled anti-human CD80 (clone 2D10; BioLegend)m or isotype-matched control antibody (Ab) for 30 min on ice. Cells were then washed, stained with 7-Amino-Actinomycin D (7-AAD; BD Biosciences) for 10 min at 4 °C in darkness, and analyzed with a FACScan flow cytometer (Becton-Dickinson, Franklin Lakes, NJ, USA), gating on viable cells (7-AAD negative cells). Data were processed using CellQuest software (Becton Dickinson). Results were expressed as mean fluorescence intensities (arithmetic means ± standard errors of the means). MHC-II expression was also assayed in the presence of a neutralizing antibody anti-IL-6 (20 µg/mL, BD Bioscience), anti-IL-10 (20 g/mL, BD Bioscience), or their isotype matched control, with 10 ng/mL of recombinant human IL-6 (rIL-6, BD Bioscience) or 10 ng/mL of recombinant human IL-10 (rIL-10, BD Bioscience) alone or plus IFN-γ used as a control.

### 4.5. Cytokine ELISA

The IL-2, IL-6, and IL-10 level were measured in culture supernatants by ELISA according to the manufacturer’s instructions (BD Biosciences).

### 4.6. Phagocytosis Assays

To study the phagocytosis capability of LX-2 cells, the phagocytic uptake of *E. coli* DH5α (Invitrogen) was measured as described [54]. Briefly, cells were infected with *B. abortus* at different MOIs, as described previously. Cells were washed twice and cultured in the presence of *E. coli* for 30 min at 37 °C in 5% CO_2_. Extracellular bacteria were washed and killed with gentamicin (100 mg/mL) for 30 min. Cells were washed, lysed with 0.1% (v/v) Triton X-100, plated overnight on tripteine soy broth (TSB) agar, and colony forming units (CFU) were counted. As a positive control, the same bacteria phagocytic test was assessed using the murine macrophage cell line J774.A1.

### 4.7. mRNA Preparation and RT-qPCR

Total cellular RNA from LX-2 cells was extracted using Quick-RNA MiniPrep Kit (Zymo Research) and 1 µg of RNA was employed to perform the reverse transcription by means of Improm-II Reverse Transcriptase (Promega). Quantitative reverse-transcription polymerase chain reaction (qRT-PCR) analysis was achieved run on a StepOne real-time PCR detection system (Life Technology) using SYBR Green as a fluorescent DNA binding dye. The conditions of the amplification reaction were the following: 10 min 95 °C, 40 cycles for 15 s at 95 °C, 58 °C for 30 s, and 72 °C for 60 s. Primer sequences used for amplification were: β-actin, forward AACAGTCCGCCTAGAAGCAC, reverse 5′-CGTTGACATCCGTAAAGACC; cathepsin-S, forward 5′-TTATGGCAGAGAAGATGTCC, reverse 5′-AAGAGGGAAAGCTAGCAATC; CIITA, forward 5′-CCGACACAGACACCATCAAC, reverse 5′-TTTTCTGCCCAACTTCTGCT. All primer sets yielded a single product of the correct size. Relative transcript levels were calculated using the ΔΔCt method using as normalizer gene β-actin.

Endpoint PCR products were subjected to electrophoresis in 1% agarose gel, stained with ethidium bromide, visualized under UV light, and photographed. In order to normalize the qRT-PCR, the β-actin gene was included as housekeeping.

### 4.8. Ag Processing and Presentation Assays

LX-2 cells were cultured in 96-well flat-bottom plates (10^5^ cells/well) and infected with *B. abortus* or stimulated with 500 U/mL of IFN-γ (Endogen) for 72 h. Following incubation and medium remotion, the cells were widely washed prior to Ag exposure. The cells then were pulsed with Ag85B (Abcam) 1, 10, and 30 µg/mL for 6 h, followed by incubation with DB1 T hybridoma cells (10^5^ cells/well). After 2 to 24 h the supernatants were harvested and the amount of interleukin-2 (IL-2) secreted by T hybridoma cells was determined by ELISA.

### 4.9. Statistical Analysis

One-way ANOVA, followed by a Post Hoc Tukey Test using GraphPad Prism 4.0 software, was used to perform the statistical analysis of the results. The obtained data were represented as mean ± SEM.

## References

[1] Pappas G., Akritidis N., Bosilkovski M., Tsianos E. (2005). Brucellosis. N. Engl. J. Med..

[2] Hayoun M.A., Smith M.E., Shorman M. (2020). Brucellosis.

[3] Kelly A.M., Golden-Mason L., Traynor O., Geoghegan J., McEntee G., Hegarty J.E., O’Farrelly C. (2006). Changes in hepatic immunoregulatory cytokines in patients with metastatic colorectal carcinoma: Implications for hepatic anti-tumour immunity. Cytokine.

[4] Robinson M.W., Harmon C., O’Farrelly C. (2016). Liver immunology and its role in inflammation and homeostasis. Cell. Mol. Immunol..

[5] Madkour M.M., Madkour M.M. (2001). Gastrointestinal brucellosis. Madkour’s Brucellosis.

[6] Akritidis N., Tzivras M., Delladetsima I., Stefanaki S., Moutsopoulos H.M., Pappas G. (2007). The liver in brucellosis. Clin. Gastroenterol. Hepatol..

[7] Heller T., Belard S., Wallrauch C., Carretto E., Lissandrin R., Filice C., Brunetti E. (2015). Patterns of hepatosplenic brucella abscesses on cross-sectional imaging: A review of clinical and imaging features. Am. J. Trop Med. Hyg..

[8] Crispe I.N. (2009). The liver as a lymphoid organ. Annu. Rev. Immunol..

[9] Arriola Benitez P.C., Scian R., Comerci D.J., Serantes D.R., Vanzulli S., Fossati C.A., Giambartolomei G.H., Delpino M.V. (2013). Brucella abortus induces collagen deposition and MMP-9 down-modulation in hepatic stellate cells via TGF-beta1 production. Am. J. Pathol..

[10] Thomson A.W., Knolle P.A. (2010). Antigen-presenting cell function in the tolerogenic liver environment. Nat. Rev. Immunol..

[11] Winau F., Hegasy G., Weiskirchen R., Weber S., Cassan C., Sieling P.A., Modlin R.L., Liblau R.S., Gressner A.M., Kaufmann S.H. (2007). Ito cells are liver-resident antigen-presenting cells for activating T cell responses. Immunity.

[12] Dunham R.M., Thapa M., Velazquez V.M., Elrod E.J., Denning T.L., Pulendran B., Grakoui A. (2013). Hepatic stellate cells preferentially induce Foxp3^+^ regulatory T cells by production of retinoic acid. J. Immunol..

[13] Roop R.M., Bellaire B.H., Valderas M.W., Cardelli J.A. (2004). Adaptation of the Brucellae to their intracellular niche. Mol. Microbiol..

[14] McCormick P.J., Martina J.A., Bonifacino J.S. (2005). Involvement of clathrin and AP-2 in the trafficking of MHC class II molecules to antigen-processing compartments. Proc. Natl. Acad. Sci. USA.

[15] Barrionuevo P., Cassataro J., Delpino M.V., Zwerdling A., Pasquevich K.A., Garcia Samartino C., Wallach J.C., Fossati C.A., Giambartolomei G.H. (2008). Brucella abortus inhibits major histocompatibility complex class II expression and antigen processing through interleukin-6 secretion via Toll-like receptor 2. Infect. Immun..

[16] Barrionuevo P., Delpino M.V., Pozner R.G., Velasquez L.N., Cassataro J., Giambartolomei G.H. (2013). Brucella abortus induces intracellular retention of MHC-I molecules in human macrophages down-modulating cytotoxic CD8(+) T cell responses. Cell. Microbiol..

[17] Mittal S.K., Roche P.A. (2015). Suppression of antigen presentation by IL-10. Curr. Opin. Immunol..

[18] Delpino M.V., Barrionuevo P., Scian R., Fossati C.A., Baldi P.C. (2010). Brucella-infected hepatocytes mediate potentially tissue-damaging immune responses. J. Hepatol..

[19] Herkel J., Jagemann B., Wiegard C., Lazaro J.F., Lueth S., Kanzler S., Blessing M., Schmitt E., Lohse A.W. (2003). MHC class II-expressing hepatocytes function as antigen-presenting cells and activate specific CD4 T lymphocyutes. Hepatology.

[20] Golden-Mason L., Douek D.C., Koup R.A., Kelly J., Hegarty J.E., O’Farrelly C. (2004). Adult human liver contains CD8pos T cells with naive phenotype, but is not a site for conventional alpha beta T cell development. J. Immunol..

[21] Giuffre M., Campigotto M., Campisciano G., Comar M., Croce L.S. (2020). A story of liver and gut microbes: How does the intestinal flora affect liver disease? A review of the literature. Am. J. Physiol. Gastrointest. Liver Physiol..

[22] Comerci D.J., Martinez-Lorenzo M.J., Sieira R., Gorvel J.P., Ugalde R.A. (2001). Essential role of the VirB machinery in the maturation of the Brucella abortus-containing vacuole. Cell. Microbiol..

[23] De Figueiredo P., Ficht T.A., Rice-Ficht A., Rossetti C.A., Adams L.G. (2015). Pathogenesis and immunobiology of brucellosis: Review of Brucella-host interactions. Am. J. Pathol..

[24] Friedman S.L. (2008). Hepatic stellate cells: Protean, multifunctional, and enigmatic cells of the liver. Physiol. Rev..

[25] Vinas O., Bataller R., Sancho-Bru P., Gines P., Berenguer C., Enrich C., Nicolas J.M., Ercilla G., Gallart T., Vives J. (2003). Human hepatic stellate cells show features of antigen-presenting cells and stimulate lymphocyte proliferation. Hepatology.

[26] Yu M.C., Chen C.H., Liang X., Wang L., Gandhi C.R., Fung J.J., Lu L., Qian S. (2004). Inhibition of T-cell responses by hepatic stellate cells via B7-H1-mediated T-cell apoptosis in mice. Hepatology.

[27] Ebrahimkhani M.R., Mohar I., Crispe I.N. (2011). Cross-presentation of antigen by diverse subsets of murine liver cells. Hepatology.

[28] Garrett W.S., Chen L.M., Kroschewski R., Ebersold M., Turley S., Trombetta S., Galan J.E., Mellman I. (2000). Developmental control of endocytosis in dendritic cells by Cdc42. Cell.

[29] Chinnadurai R., Grakoui A. (2010). B7-H4 mediates inhibition of T cell responses by activated murine hepatic stellate cells. Hepatology.

[30] Charles R., Chou H.S., Wang L., Fung J.J., Lu L., Qian S. (2013). Human hepatic stellate cells inhibit T-cell response through B7-H1 pathway. Transplantation.

[31] Reith W., LeibundGut-Landmann S., Waldburger J.M. (2005). Regulation of MHC class II gene expression by the class II transactivator. Nat. Rev. Immunol..

[32] Jabrane-Ferrat N., Nekrep N., Tosi G., Esserman L., Peterlin B.M. (2003). MHC class II enhanceosome: How is the class II transactivator recruited to DNA-bound activators?. Int. Immunol..

[33] Gobin S.J., Peijnenburg A., Keijsers V., van den Elsen P.J. (1997). Site alpha is crucial for two routes of IFN gamma-induced MHC class I transactivation: The ISRE-mediated route and a novel pathway involving CIITA. Immunity.

[34] Gobin S.J., Peijnenburg A., van Eggermond M., van Zutphen M., van den Berg R., van den Elsen P.J. (1998). The RFX complex is crucial for the constitutive and CIITA-mediated transactivation of MHC class I and beta2-microglobulin genes. Immunity.

[35] Gobin S.J., van Zutphen M., Westerheide S.D., Boss J.M., van den Elsen P.J. (2001). The MHC-specific enhanceosome and its role in MHC class I and beta(2)-microglobulin gene transactivation. J. Immunol..

[36] Reyes V.E., Lu S., Humphreys R.E. (1991). Cathepsin B cleavage of Ii from class II MHC alpha- and beta-chains. J. Immunol..

[37] Bevec T., Stoka V., Pungercic G., Dolenc I., Turk V. (1996). Major histocompatibility complex class II-associated p41 invariant chain fragment is a strong inhibitor of lysosomal cathepsin L.. J. Exp. Med..

[38] Fineschi B., Sakaguchi K., Appella E., Miller J. (1996). The proteolytic environment involved in MHC class II-restricted antigen presentation can be modulated by the p41 form of invariant chain. J. Immunol..

[39] Riese R.J., Mitchell R.N., Villadangos J.A., Shi G.P., Palmer J.T., Karp E.R., De Sanctis G.T., Ploegh H.L., Chapman H.A. (1998). Cathepsin S activity regulates antigen presentation and immunity. J. Clin. Investig..

[40] Nakagawa T., Roth W., Wong P., Nelson A., Farr A., Deussing J., Villadangos J.A., Ploegh H., Peters C., Rudensky A.Y. (1998). Cathepsin L: Critical role in Ii degradation and CD4 T cell selection in the thymus. Science.

[41] Maubach G., Lim M.C., Kumar S., Zhuo L. (2007). Expression and upregulation of cathepsin S and other early molecules required for antigen presentation in activated hepatic stellate cells upon IFN-gamma treatment. Biochim. Biophys. Acta.

[42] Arriola Benitez P.C., Pesce Viglietti A.I., Gomes M.T.R., Oliveira S.C., Quarleri J.F., Giambartolomei G.H., Delpino M.V. (2019). Brucella abortus infection elicited hepatic stellate cell-mediated fibrosis through inflammasome-dependent IL-1beta production. Front. Immunol..

[43] Gomes M.T., Campos P.C., Oliveira F.S., Corsetti P.P., Bortoluci K.R., Cunha L.D., Zamboni D.S., Oliveira S.C. (2013). Critical role of ASC inflammasomes and bacterial type IV secretion system in caspase-1 activation and host innate resistance to Brucella abortus infection. J. Immunol..

[44] Gorvel J.P., Moreno E. (2002). Brucella intracellular life: From invasion to intracellular replication. Vet. Microbiol..

[45] Kohler S., Michaux-Charachon S., Porte F., Ramuz M., Liautard J.P. (2003). What is the nature of the replicative niche of a stealthy bug named Brucella?. Trends Microbiol..

[46] Zhan Y., Cheers C. (1993). Endogenous gamma interferon mediates resistance to Brucella abortus infection. Infect. Immun..

[47] Dornand J., Gross A., Lafont V., Liautard J., Oliaro J., Liautard J.P. (2002). The innate immune response against Brucella in humans. Vet. Microbiol..

[48] Schroder K., Hertzog P.J., Ravasi T., Hume D.A. (2004). Interferon-gamma: An overview of signals, mechanisms and functions. J. Leukoc. Biol..

[49] Hop H.T., Reyes A.W.B., Huy T.X.N., Arayan L.T., Min W., Lee H.J., Rhee M.H., Chang H.H., Kim S. (2018). Interleukin 10 suppresses lysosome-mediated killing of Brucella abortus in cultured macrophages. J. Biol. Chem..

[50] Giambartolomei G.H., Scian R., Acosta-Rodriguez E., Fossati C.A., Delpino M.V. (2012). Brucella abortus-infected macrophages modulate T lymphocytes to promote osteoclastogenesis via IL-17. Am. J. Pathol..

[51] Kitamura H., Kamon H., Sawa S., Park S.J., Katunuma N., Ishihara K., Murakami M., Hirano T. (2005). IL-6-STAT3 controls intracellular MHC class II alphabeta dimer level through cathepsin S activity in dendritic cells. Immunity.

[52] Fumeaux T., Pugin J. (2002). Role of interleukin-10 in the intracellular sequestration of human leukocyte antigen-DR in monocytes during septic shock. Am. J. Respir. Crit. Care Med..

[53] Scian R., Barrionuevo P., Giambartolomei G.H., De Simone E.A., Vanzulli S.I., Fossati C.A., Baldi P.C., Delpino M.V. (2011). Potential role of fibroblast-like synoviocytes in joint damage induced by Brucella abortus infection through production and induction of matrix metalloproteinases. Infect. Immun..

[54] Gaikwad S., Agrawal-Rajput R. (2015). Lipopolysaccharide from rhodobacter sphaeroides attenuates microglia-mediated inflammation and phagocytosis and directs regulatory T cell response. Int. J. Inflam..

